# Exploration of the B3 transcription factor superfamily in *Aquilaria sinensis* reveal their involvement in seed recalcitrance and agarwood formation

**DOI:** 10.1371/journal.pone.0294358

**Published:** 2023-11-16

**Authors:** Yue Jin, Lin Zeng, Mengjun Xiao, Yanan Feng, Zhihui Gao, Jianhe Wei

**Affiliations:** 1 Key Laboratory of Bioactive Substances and Resources Utilization of Chinese Herbal Medicine, Ministry of Education & National Engineering Laboratory for Breeding of Endangered Medicinal Materials, Institute of Medicinal Plant Development, Chinese Academy of Medical Sciences and Peking Union Medical College, Beijing, China; 2 Hainan Provincial Key Laboratory of Resources Conservation and Development of Southern Medicine & Key Laboratory of State Administration of Traditional Chinese Medicine for Agarwood Sustainable Utilization, Hainan Branch of the Institute of Medicinal Plant Development, Chinese Academy of Medical Sciences and Peking Union Medical College, Haikou, China; Central University of Punjab, INDIA

## Abstract

The endangered tree species of the *Aquilaria* genus produce agarwood, a high value material produced only after wounding; however, conservation of *Aquilaria* seeds is difficult. The B3 transcription factor family has diverse important functions in plant development, especially in seed development, although their functions in other areas, such as stress responses, remain to be revealed. Here germination tests proved that the seeds of *A*. *sinensis* were recalcitrant seeds. To provide insights into the B3 superfamily, the members were identified and characterized by bioinformatic approaches and classified by phylogenetic analysis and domain structure. In total, 71 members were identified and classified into four subfamilies. Each subfamily not only had similar domains, but also had conserved motifs in their B3 domains. For the seed-related LAV subfamily, the B3 domain of AsLAV3 was identical to that of AsVALs but lacked a typical zf-CW domain such as VALs. AsLAV5 lacks a typical PHD-L domain present in *Arabidopsis* VALs. qRT-PCR expression analysis showed that the LEC2 ortholog AsLAV4 was not expressed in seeds. RAVs and REMs induced after wound treatment were also identified. These findings provide insights into the functions of *B3* genes and seed recalcitrance of *A*. *sinensis* and indicate the role of *B3* genes in wound response and agarwood formation.This is the first work to investigate the B3 family in *A*. *sinensis* and to provide insights of the molecular mechanism of seed recalcitrance.This will be a valuable guidance for studies of B3 genes in stress responses, secondary metabolite biosynthesis, and seed development.

## 1. Introduction

The *Aquilaria* genus includes endangered tree species in Asia that can produce a high-valued non-timber material known as agarwood. Agarwood is widely used as a traditional medicine, perfume, and incense in Asian countries [1]. It is only formed when trunks, branches, or roots are injured and various of sesquiterpenes and 2-(2-phenylethyl) chromones accumulate at the injured sites [2]. The limited yield of agarwood in wild *Aquilaria* trees and over-harvesting has led to the serious damage of natural resource of this genus. Currently, all species in this genus are listed on the International Union for Conservation of Nature red list as endangered species [3] and are on the Appendix II list of the Convention on International Trade in Endangered Species of Wild Fauna and Flora [4]. Aquilaria seedlings are mainly obtained by seed germination. However, we have found that the seeds of *A*. *sinensis*, a species native in China, are difficult to conserve and protect, which might be caused by the low tolerance to water loss.

The B3 transcription factor (TF) superfamily is a plant-specific family that contains a B3 DNA-binding domain of approximately 110 amino acids (aa). According to the types of these domains, the B3 superfamily could be classified into four gene families, the LEAFY COTYLEDON2-ABSCISIC ACID INSENSITIVES3-VAL (LAV), ARF, RELATED TO ABI3 and VP1 (RAV), and REPRODUCTIVE MERISTEM (REM) families [5]. The LAV family members can be further classified into the AFL and VAL subfamilies. The B3 domains from different families can bind to distinct DNA sites [6]. For example, the B3 domain of the ABSCISIC ACID INSENSITIVE 3 (ABI3) proteins recognizes the Sph/RY element of the CATGCA sequence [7–9]. The C-terminal B3 domain of some proteins of the RAV family recognizes the CACCTG sequence [10].

A series of B3 TFs have been shown to regulate a wide range of biological processes in the plant life cycle [6, 11–13], especially in seed development. The AFL subfamily plays a central role in seed development, including seed maturation, desiccation tolerance, and germination [14]. AFL members and their antagonistic VAL repressors from the same LAV family control the developmental transition from seed to seedling. The RAV family, with more members in *Arabidopsis*, has been demonstrated to have functions in diverse biological processes, including seed germination and abiotic stresses. The ARF family is a widely investigated family in *Arabidopsis*. Members of this family have distinct or redundant functions and regulate almost every aspect of plant growth and development [15]. Several studies reported that the B3 superfamily members may participate in secondary metabolism. RAV1 (an AP2/B3 domain TF) was shown to regulate the *HMGR* gene as well as epoxysqualene biosynthesis and sterol biosynthesis in *Arabidopsis* [16]; furthermore, flavonoid biosynthesis was influenced by the AFL member, AtLEC2 [17]. A study found that two JA-responsive AP2 family TFs (AaERF1 and AaERF2-from *Artemisia annua* L.) were two positive regulators of artemisinin biosynthesis [18]. These results indicated the possible roles of B3 TFs in plant secondary metabolism.

Therefore, this study focused on the genome-wide identification and investigation of B3 TFs in *A*. *sinensis* to provide insights on their evolution and their functions in wound-induced agarwood formation and seed recalcitrance. This is the first work to investigate the B3 family in *A*. *sinensis* and the first effort to provide insights of the molecular mechanism of seed recalcitrance. We found that each subfamily not only had similar domains, but also had conserved motifs in their B3 domains. For the seed-related LAV subfamily, special characteristics of their domains were identified. The seed-gernination-related LEC2 ortholog AsLAV4 was not expressed in seeds. Furthermore, four RAVs and three REMs induced at 21 months after wound treatment were also identified, demonstrating the involvement of B3 genes in wound response and agarwood formation. This will be a valuable guidance for studies of B3 genes in stress responses, secondary metabolite biosynthesis, and seed development.

## 2. Results

### 2.1. Genome-wide identification and characterization of *B3* genes in *A*. *sinensis*

The Hidden Markov Model (HMM) profile of the Pfam B3 domain was used to identify 71 candidate *B3* family genes in *A*. *sinensis*. After confirmation of each AsB3 protein using the SMART database and National Center for Biotechnology Information Conserved Domains (CD)-search tools, the online program ProtParam was used to analyze the molecular characteristics of each predicted protein, including the amino acid length, theoretical isoelectric point (pI), and the molecular weight (S1 Table). B3 proteins ranged from 92 to 1,138 aa in size; 47 proteins were <400 aa, and only 19 members were > 500 aa. The molecular weights of these proteins were also divergent and ranged from 10,779.27 to 126,017.29 Da. The pI values ranged from 5.24 to 10.57. The predicted subcellular localizations of AsB3 proteins indicated their presence in different organelles: 35 proteins were located in nucleus, 26 proteins were located in the cytoplasm, 9 proteins were located in mitochondria, one REM protein was located in the extracellular environment. Most proteins predicted to be in the cytoplasm belong to the RAV or REM subfamilies, and their lengths were comparatively short.

### 2.2. Phylogenetic analysis and classification of *AsB3* genes

**Fig 1:**
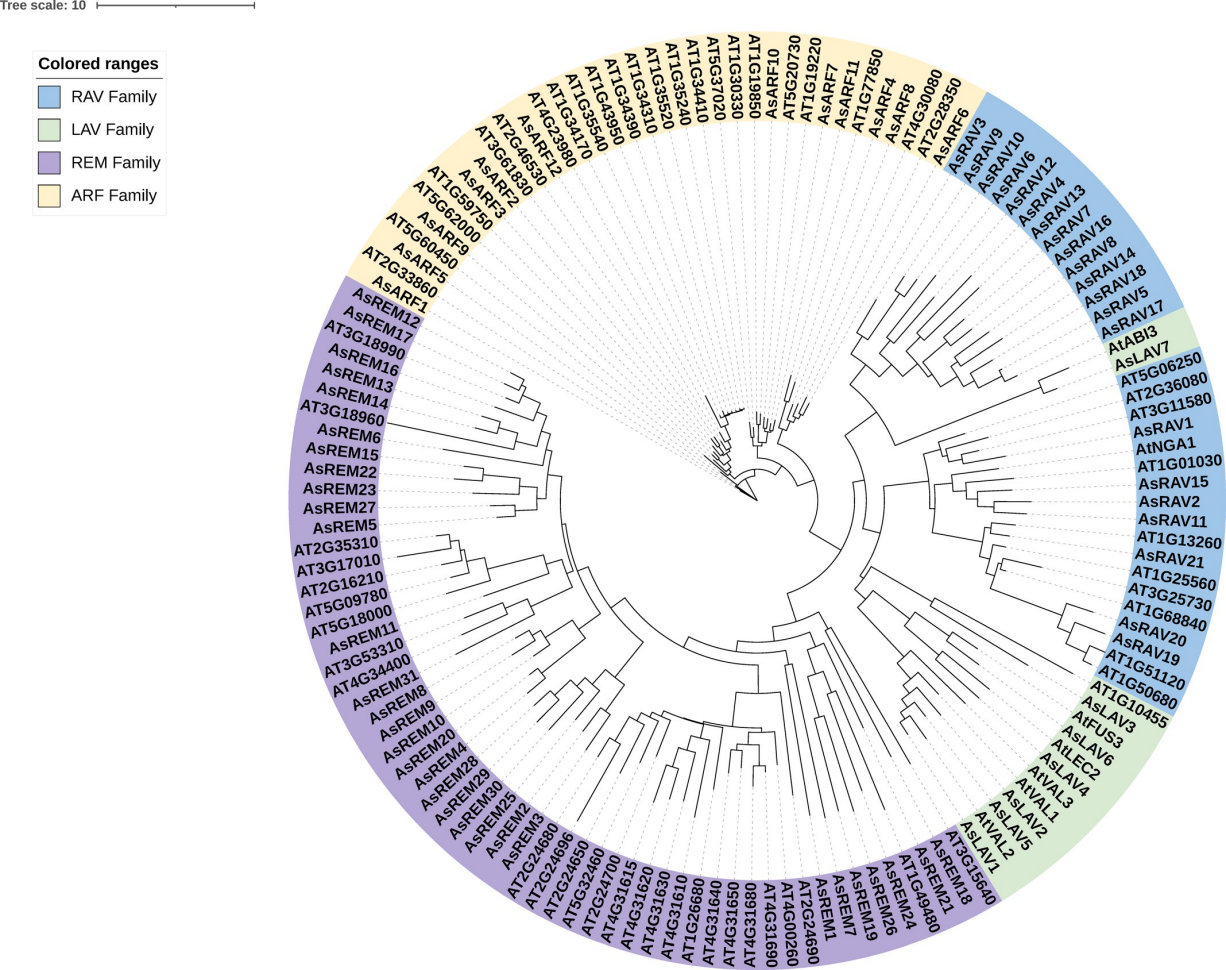
Phylogenetic tree of B3 proteins from *A*. *sinensis* and *Arabidopsis*. The phylogenetic tree was constructed with the IQ-TREE program by the maximum likelihood method.

To classify the AsB3s, a phylogenetic tree was constructed via IQ-TREE using the protein sequences of all AsB3s, and 68 Arabidopsis B3 protein sequences were used as reference proteins (S2 Table). The classification showed that AsLAV5, AsLAV1, and AsLAV2 were closely related to VAL1, VAL2, and VAL3 in *Arabidopsis*, respectively (Fig 1, S1 Fig). AsLAV6 was closely related to FUS3 in *Arabidopsis*. AsLAV7 was closely related to ABI3 in *Arabidopsis*. AsLAV4 also clustered with the AFL subfamily and was closely related to LEC2 (S1 Fig). AsLAV3 did not cluster with the AFL or VAL group. For the RAV subfamily, 21 members were present in the large clade with the Arabidopsis NGA1; Among them, AsRAV2, AsRAV11, and AsRAV15 were the most closely related to NGA1 (At2g46870). All 12 AsARFs clustered together, and the remaining 31 B3 members were in the large REM clade. Interestingly, a sub-clade of only *A*. *sinensis* RAVs (AsRAV3-10, etc.) and a sub-clade of only *Arabidopsis* REMs (At2g24680, etc.) were found in the phylogenetic tree. Blasting of these AsRAVs in NCBI showed that they all have low sequence identity with other protein sequences in NCBI NR database, demonstrating that they were unique in *A*. *sinensis*.

### 2.3. Gene structure and conserved motif of the *AsB3* genes

**Fig 2:**
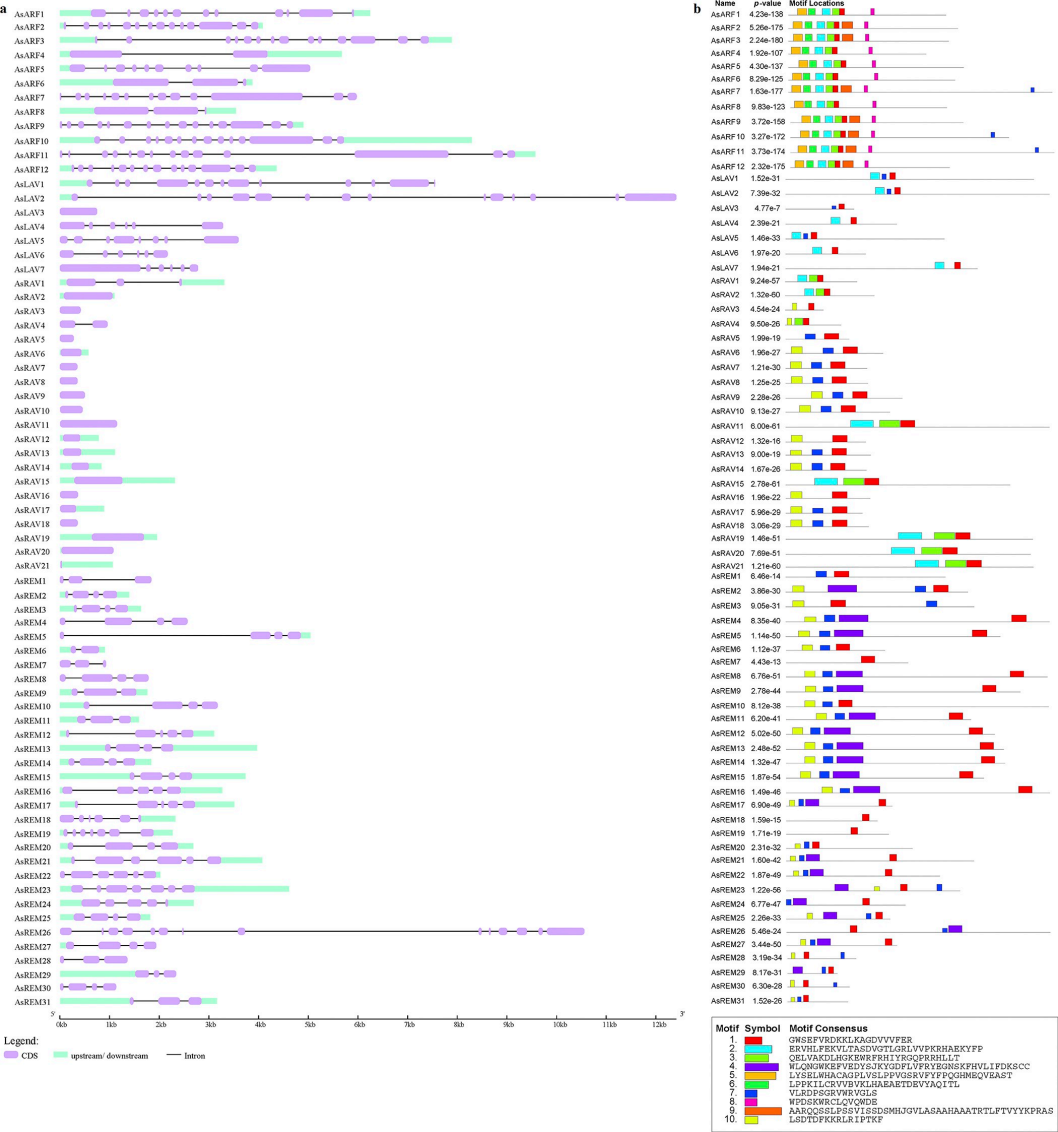
Gene structure analysis of 71 B3 genes (a) and conserved motif analysis of B3 proteins (b) in *A*. *sinensis*. For gene structures, exons are represented by purple boxes, and introns are represented by black lines. For conserved motifs, each motif is represented by a colored box, and the box length corresponds to the motif length.

The exon numbers were diverse in both the *B3* family and subfamily. For the *LAV* subfamily, the *AFL* members *AsLAV4*, *AsLAV6*, and *AsLAV7* contained six exons (Fig 2A), with the first exon of *AsLAV7* being much longer than the other exons. *AsLAV3* contained one exon only. All *VAL* members had more exons. *AsLAV5*, *AsLAV1*, and *AsLAV2* had 8, 13, and 14 exons, respectively. The *RAV* subfamily members only had 1–3 exons, and 19 members had only one exon. *AsRAV4* and *AsRAV1* had two and three exons, respectively. Most of the 12 *ARF* members had 12–15 exons, whereas *AsARF4* had only two exons. Both *AsARF6* and *AsARF8* had three exons, and *AsARF10* had 10 exons. Except for *AsREM26*, which had 14 exons, all *REM* members had <10 exons, and most of them had 3–6 exons.

Analysis of the conserved motifs showed that all B3 members of *A*. *sinensis* contained 1–3 B3 domains (S1 Table). All the seven LAV family members had a single B3 domain while the three VAL members in them also had one zf-CW domain. Each RAV member also had only a single B3 domain, and three (AsRAV19, AsRAV20, and AsRAV21) possessed an AP2 domain. All the 12 ARF members had one B3 domain and one Auxin_resp domain while seven of them also had an AUX_IAA domain; AsARF2 had two AUX_IAA domains. The REM family only contained B3 domains, but the numbers of this domain differed from one to three. Except for AsREM24, which had a 60-aa B3 domain, the lengths of all B3 domains in B3 proteins were close to 100 aa. All zf-CW domains contained 44 aa, whereas all the Auxin_resp domains contained 80 aa. We also found that newly identified *A*. *sinesis*-specific RAVs (AsRAV3-10, 12–14,16–18) had similar motif structures, compared with other AsRAVs (Fig 2B).

To gain more insights into the structure of B3 proteins in *A*. *sinensis*, sequence logos were produced to examine how motifs conserved (Fig 2B). Motifs 2, 7, and 1 were sequentially arranged in the B3 domain of three VAL orthologs (AsLAV1, AsLAV2, and AsLAV5), and three AFLs (AsLAV4, AsLAV6, and AsLAV7) had motifs 2 and 1 in the B3 domain. The motif components of the B3 domain in AsLAV3 were similar to those in the VAL subgroup. However, the p-values of the three motifs in AsLAV3 were higher than those in the three VAL members, demonstrating a lower possibility. Considering that the AsLAV3 does not contain the zf-CW domain, these results could help to explain why the AsLAV3 in the phylogenetic tree was clustered neither in the AFL nor in the VAL subgroup. Motifs 2, 3, and 1 were sequentially arranged in the B3 domain of the RAV subfamily member AsRAV1, 2, 11, 15, 19, 20, and 21. Motifs 10, 7, and 1 were sequentially arranged in the B3 domain of other RAVs with some exceptions which lost the first or second motif.

Fourteen members of the REM family contained motifs 10, 7, and 4 in the first B3 domain and motif 1 in the second B3 domain. AsREM6, AsREM10, AsREM20, and AsREM31 contained motifs 10, 7, and 1 in their single B3 domains similar with RAVs. However, no conserved motif was identified in their second B3 domains except for AsREM6. Other REMs with a single B3 domain were AsREM7, AsREM18, and AsREM19 and contained only motif 1.

The motif analysis showed that the ARF family were highly conserved. Six motifs, including motif 5, 6, 2, 3, 1, and 8, were shared by all AsARFs. Motifs 5, 6, 8, and 9 were identified only in ARFs and motifs 8 and 9 were partially located in the Auxin_resp domain. Motifs 2, 3, and 1, which were located on the B3 domain, also existed in some RAV members such as AsRAV1 and AsRAV2.

Thus, the B3 proteins clustered together in the phylogenetic tree not only have similar entire sequence, but also have similar B3 domain structure. In *Arabidopsis*, the AFL repressors VAL1 and VAL2 both have a PHD-L domain, whereas VAL3 has an incomplete PHD-L domain [19]. PHD and CW-Zf domains were identified as the histone modification readers that recognize the H3K4 me3 mark and repress target gene transcription [20, 21]. We therefore investigated the PHD domain in *A*. *sinensis* and found that the VAL member AsLAV5 lacked the entire PHD-L domain (S3 Fig).

### 2.4. Chromosomal location of *B3* genes in the *A*. *sinensis* genome and gene duplication

**Fig 3:**
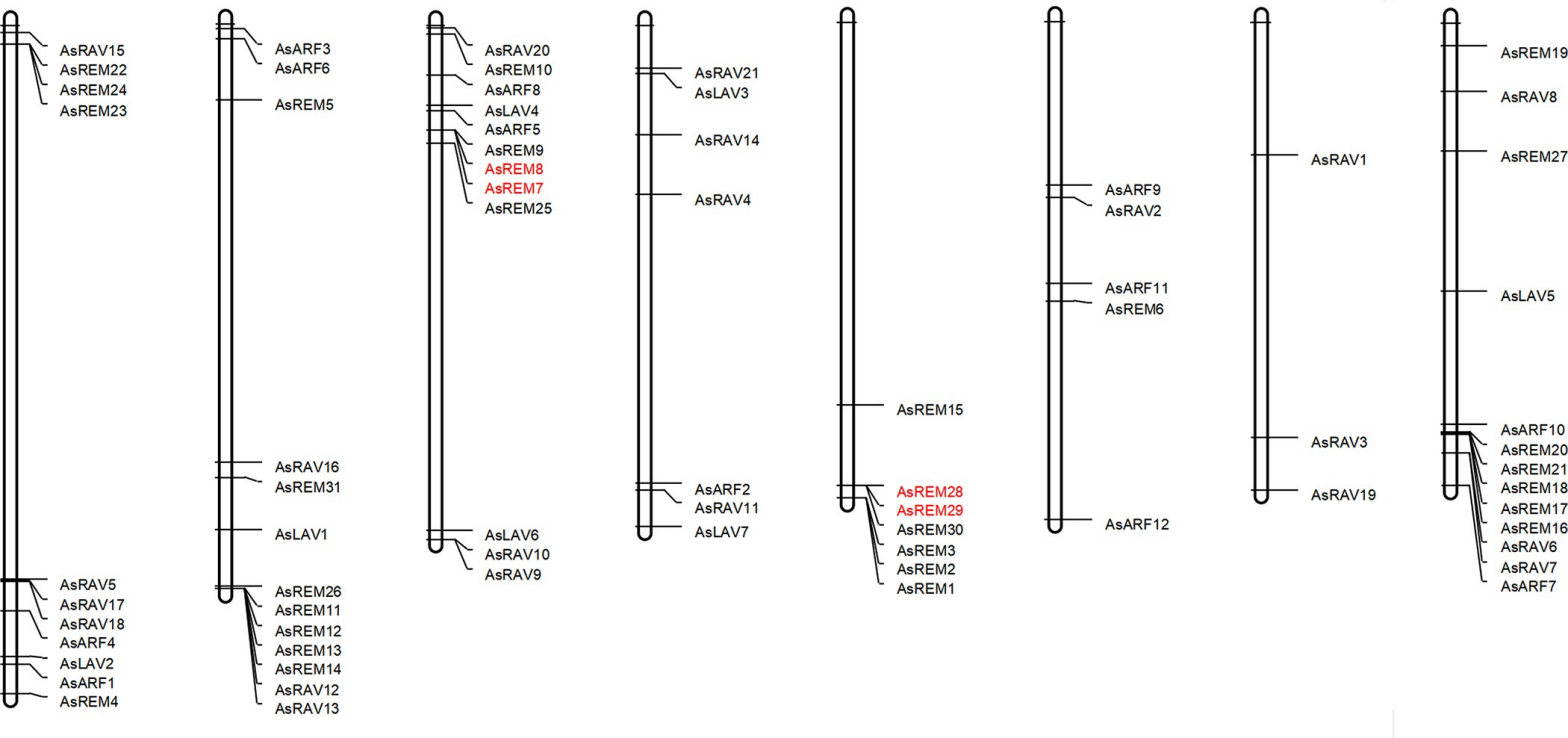
Distribution of 71 *B3* genes in the chromosomes of *A*. *sinensis*. The tandem duplication genes are indicated in red.

The chromosomal location of *B3* genes in the *A*. *sinensis* genome showed that the 71 genes could be mapped onto the eight chromosomes (Fig 3). Both Lachesis_group1 and Lachesis_group 7 contained 13 *B3* genes, which are the most of all chromosomes. However, Lachesis_group 6 had only three *B3* genes, which is the least of all chromosomes. Twelve *ARF* genes were found on six chromosomes. Lachesis_group 4 and 6 had no *ARF* genes. Other chromosomes had 1–3 *ARF*s. Lachesis_group 4 only had seven *REM*s, whereas Lachesis_group 6 only had three *RAV*s. Furthermore, we found that the *REM* genes tend to cluster together and the REM clusters were found in five chromosomes.

By the gene duplication analysis, we found two tandem duplication events: *AsREM7* & *AsREM8* and *AsREM28* & *AsREM29* (Fig 3).

### 2.5. Expression patterns of *AsB3* genes in different tissues

**Fig 4:**
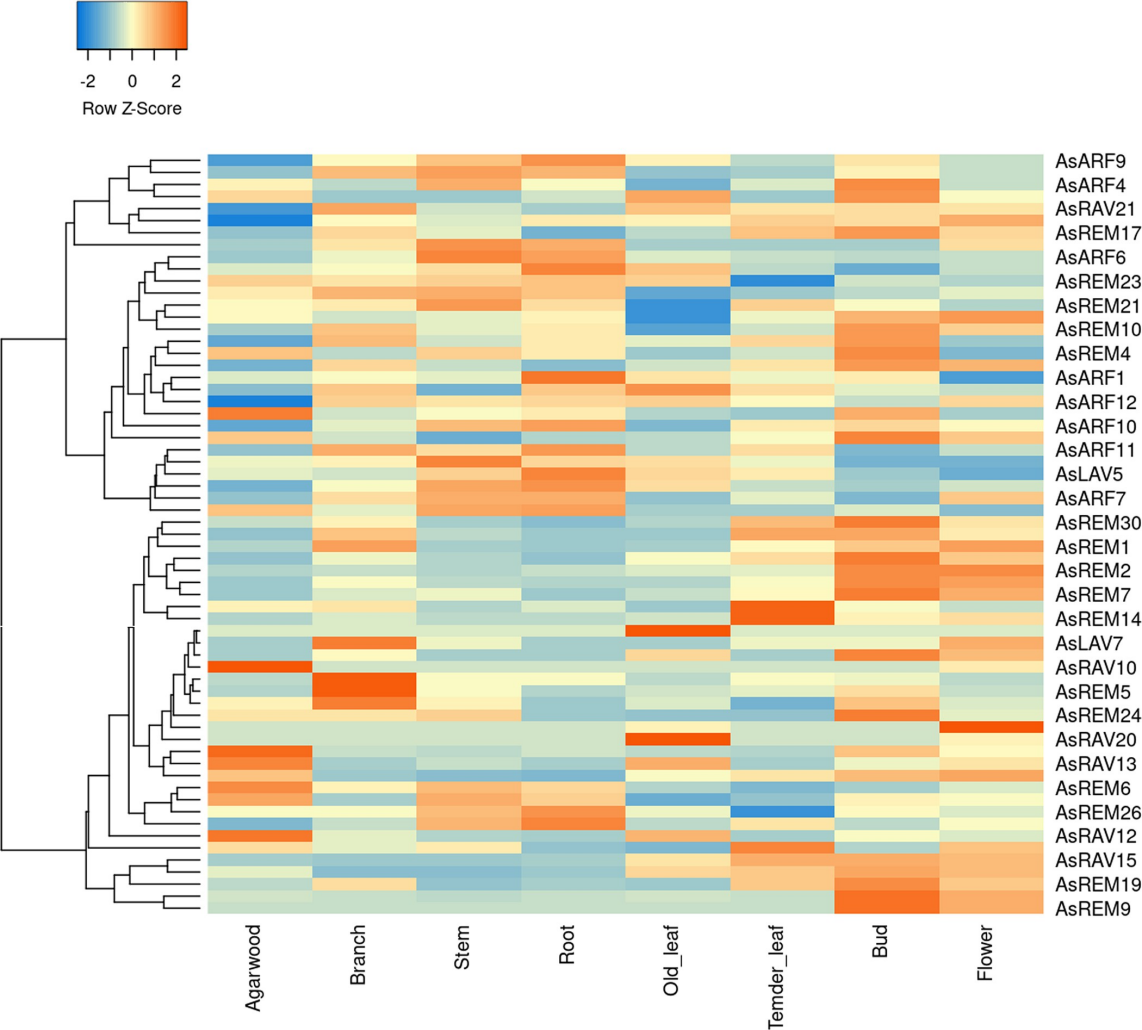
Expression profiles of *B3* genes in eight tissues of *A*. *sinensis*. All gene expression levels were transformed into scores ranging from −2 to 2. For each row, blue and orange correspond to low and high values of expression, respectively.

The RNA-seq analysis showed that *AsLAV4*, which is the ortholog of the *LEC2*, and the seven *RAV* family genes *AsRAV3*, *AsRAV5*, *AsRAV7*, *AsRAV9*, and *AsRAV16–18*, were not expressed in any of the tested tissues (Fig 4). All genes in *ARF* family were expressed, and their expression levels were comparatively higher than those of genes in other families. Furthermore, the *ARF* family genes tended to have similar expression patterns and clustered together in the heatmap. *ARF* genes and several genes from other families accumulated mainly in vegetable tissues, including the branch, stem, root, and bud. *VAL* genes *AsLAV1*, *AsLAV2*, and *AsLAV5* in the *LAV* subfamily are examples of these genes, although they were only expressed in stems and roots. All the *AFL* genes were expressed at extremely low levels in all tested tissues. *AsRAV10*, *AsREM6*, *AsRAV2*, and *AsRAV12* were expressed in agarwood. The expression patterns of the 21 members in the *RAV* family were similar to those of *AFL* members. Most of the *RAV* genes were expressed at a low level or even not expressed. Only *AsRAV1*, *AsRAV4*, *AsRAV11*, *AsRAV12*, *AsRAV14*, and *AsRAV15* were expressed in some tissues, and their expression patterns were diverse. For example, *AsRAV1* was mainly expressed in fresh leaves, *AsRAV14* was mainly expressed in the stems and roots, and *AsRAV11* and *AsRAV15* were mainly expressed in fresh leaves, flowers, and buds.

### 2.6. Seed recalcitrance and expression patterns of AFL member

**Fig 5:**
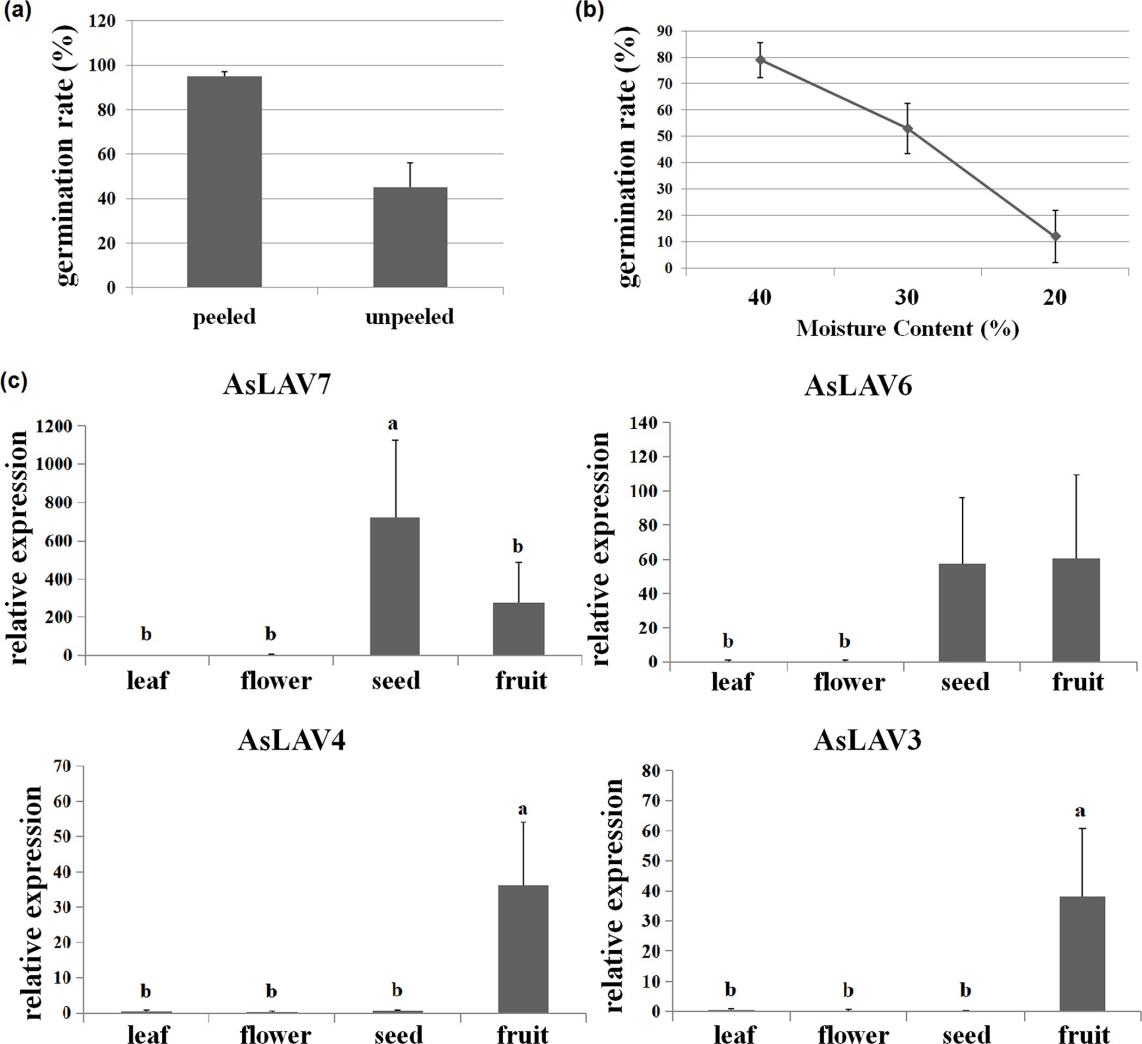
Recalcitrance of *A*. *sinensis* seeds, and expression patterns of candidate *AFL* genes in different tissues. **(a)** Germination rate of peeled and unpeeled seeds; **(b)** germination rate of seeds after desiccation treatment; and **(c)** expression pattern of candidate *AFL* genes in different tissues assessed via qRT-PCR. AsLAV7 is the ortholog of ABI3, AsLAV6 is the ortholog of FUS3, and AsLAV4 is the ortholog of LEC2. Significant analysis was performed by SPSS software using two-tailed unpaired Student’s t-tests (** indicates p< 0.01), or one-way analysis of variance (ANOVA) showed with lower cases a, b, and c in figure. Different letters indicate significant differences between two samples.

On receipt of the seeds, we compared the germination rate of peeled and unpeeled seeds. Almost 95% of peeled seeds germinated while only 45% of unpeeled seeds germinated (Fig 5A). Then, we peeled all the tested seeds for the following experiments. Fresh seeds with a moisture content of 40% had a 79% germination rate and produced healthy seedlings. After drying, the germination rate quickly declined to 53% and 12% at the moisture contents of 30% and 20%, respectively (Fig 5B). The loss of viability showed the typical pattern found in other recalcitrant seeds, demonstrating that they are recalcitrant seeds.

To provide molecular insight into the low desiccation tolerance of the seeds, we used qRT-PCR to examine the expression level of the seed-related *AFL* subfamily genes in seeds, leaves, flowers, and fruits. The results showed that the *ABI3* ortholog *AsLAV7* and *FUS3* ortholog *AsLAV6* were expressed in seeds and fruits, whereas the *LEC2* ortholog *AsLAV4* was expressed only in fruits (Fig 5C); *AsLAV3* was expressed only in fruits.

### 2.7. Expression pattern of *AsB3* genes after wound treatment

**Fig 6:**
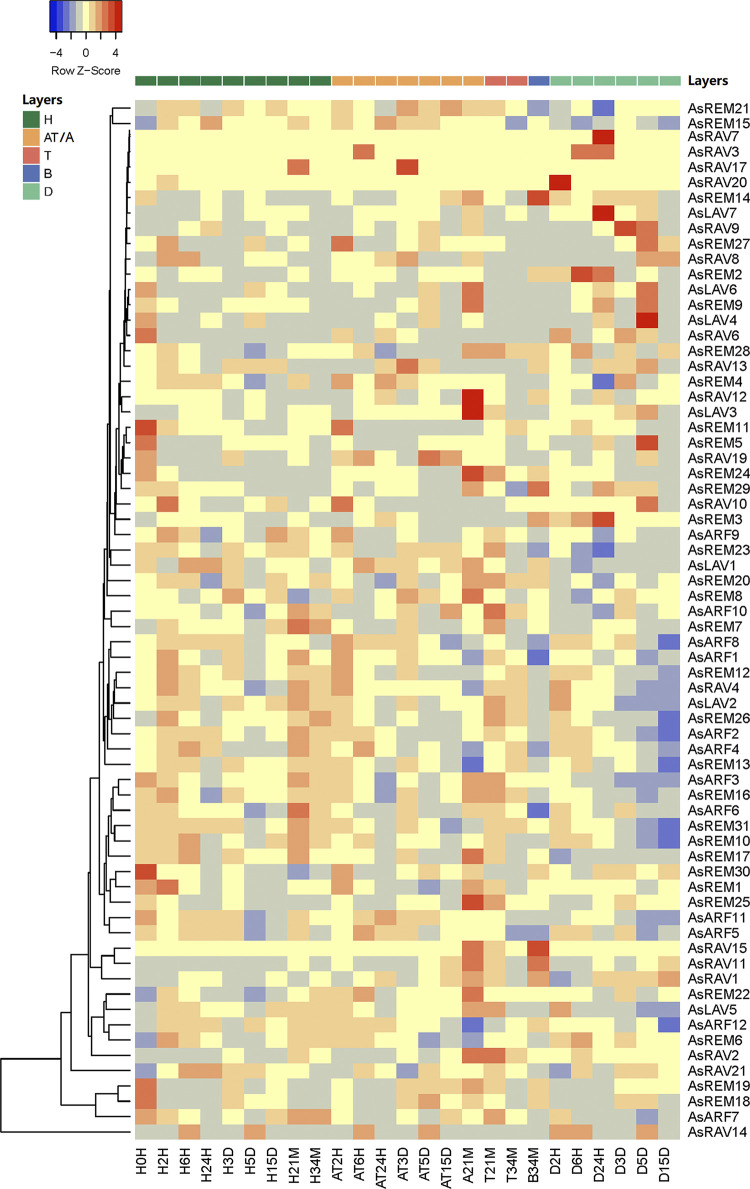
Expression profiles of *B3* genes in different layers at 2, 6, and 24 h, 3, 5, and 15 d, and 21, and 34 m after wound treatment. H, healthy layer; A, agarwood layer; T, transition layer; B, blocked layer; D, dead layer after Agar-Wit treatment; AT, agarwood and transition layer (at the early timepoints, the agarwood layer and transition layer could not be clearly divided). The expression data of all wound-treated samples were standardized and then processed to generate the Heat Map with Heatmapper with the y = log_2_ + [(Trpkm + 1)/(Crpkm + 1)] as the ordinate, Trpkm indicates the rpm value of the treated group, and Crpkm indicates the rpm value of the control group H0H (untreated sample, H layer at 0 h after treatment). All gene expression levels were transformed into scores ranging from −4 to 4. For each row, blue and orange correspond to low and high values of expression, respectively.

We then examined the expression pattern of *AsB3* genes to identify potential agarwood-related *B3* genes. The *RAV* genes *AsRAV16*, *AsRAV5*, and *AsRAV18* were not expressed in all these wound-treated tissues (Fig 6). Except for *AsARF12*, the expression patterns of other *ARF* genes tended to be similar. *AFL* genes *AsLAV7*, *AsLAV6*, *AsLAV4*, and the unclassified *AsLAV3* clustered closely in the heatmap and tended to be expressed in the dead layer (D layer). However, *AsLAV5* clustered far from *AsLAV1* and *AsLAV2*, demonstrating that the expression patterns of *VAL* members were less similar to each other. The expression pattern of *AsLAV3* was similar to that of *AFL* family members in different tissues and in the wound-treated stems. *AsRAV2* and *AsRAV12*, which were expressed in agarwood, were also expressed in the A21M sample. We found that at 21 months after wound treatment, a series of *B3* TFs was induced, which is consistent with the expression patterns of terpene synthase genes (unpublished results). For example, the expression of *AsRAV1*, *AsRAV2*, *AsRAV11*, *AsRAV15* and *AsREM8*, *AsREM15*, and *AsREM17* was induced in the A21M sample. Among them, AsRAV1 and AsRAV15 were two enzymes that clustered closely to the AtRAV1 in the phylogenetic tree (Fig 1). The *A*. *sinensis*-specified *AsRAV12* not only expressed in agarwood tissue (Fig 4), but also expressed in A21M sample (Fig 6). While some of the unique RAVs in *A*. *sinensis*, for example, *AsRAV3*,*17* also expressed in agarwood layers at different timepoints, although they were not expressed in different tissues (Fig 4). These demonstrated that these expanded members might have some functions in agarwood formation.

## 3. Discussion

Overall, 71 *B3* superfamily genes, including 7 *LAV*s, 21 *RAV*s, 12 *ARF*s, and 31 *REM*s, were identified in the genome of *A*. *sinensis*. For the *LAV* family, the orthologs of all three *AFL* subfamily members and three *VAL* subfamily members in *Arabidopsis* were identified using phylogenetic analysis. These members shared the characteristics of *LAV* families in other plants [14, 22, 23]. For example, AFL members possess a single B3 domain, and VAL members possess B3 and zf-CW domains. This demonstrated that the LAV family was highly conserved. Three domains (B3, Auxin_resp, AUX_IAA) were found in the AsARF family, and this is consistent with studies in grape and tobacco [22, 23]. However, in tobacco, only two ARF proteins have the B3 domain, whereas in *A*. *sinensis* and grape, all ARF members have both B3 and Auxin_resp domains. The study in grape identified an AP2 domain in some RAV members, which is consistent with our findings. Similarly, a series of REM members contained multiple B3 domains, but the highest number of B3 domains in *A*. *sinensis* was less than that of grape [23] and tobacco [22].

According to a previous study, the organization of genes in *ABI3/VP1* clade is homogeneous: 5–7 exons and 3–5 introns [14]. In *A*. *sinensis*, the three *AFL* genes follow this rule and are even more conserved because they all have six exons and five introns. However, this result is not consistent with that in tobacco, which showed that most *LAV* genes have >10 exons. We also found that the first exon of *AsLAV7*, which is the ortholog of *ABI3*, was much longer than other exons. This is consistent with previous reports [14]. Furthermore, the *VAL* genes were less conserved compared with the *AFL* genes. This result is not consistent with a previous study in grape showed a more conserved organization in the *VAL* subfamily than that in the *AFL* subfamily [23]. This demonstrated that the gene structures of the *LAV* family lack conservation among different species. The *ARF* subfamily is more conserved in the exon numbers in *A*. *sinensis* than in grape and tobacco [22, 23]. Similar to our results, the *RAV* genes contain fewer exons in grape and tobacco. The exon numbers of the *REM* subfamily in *A*. *sinensis* were diverse, which also agrees with findings in grape and tobacco. *AsREMs* tended to be clustered in the genome, which is similar to results from with previous studies [13, 22].

**Fig 7:**
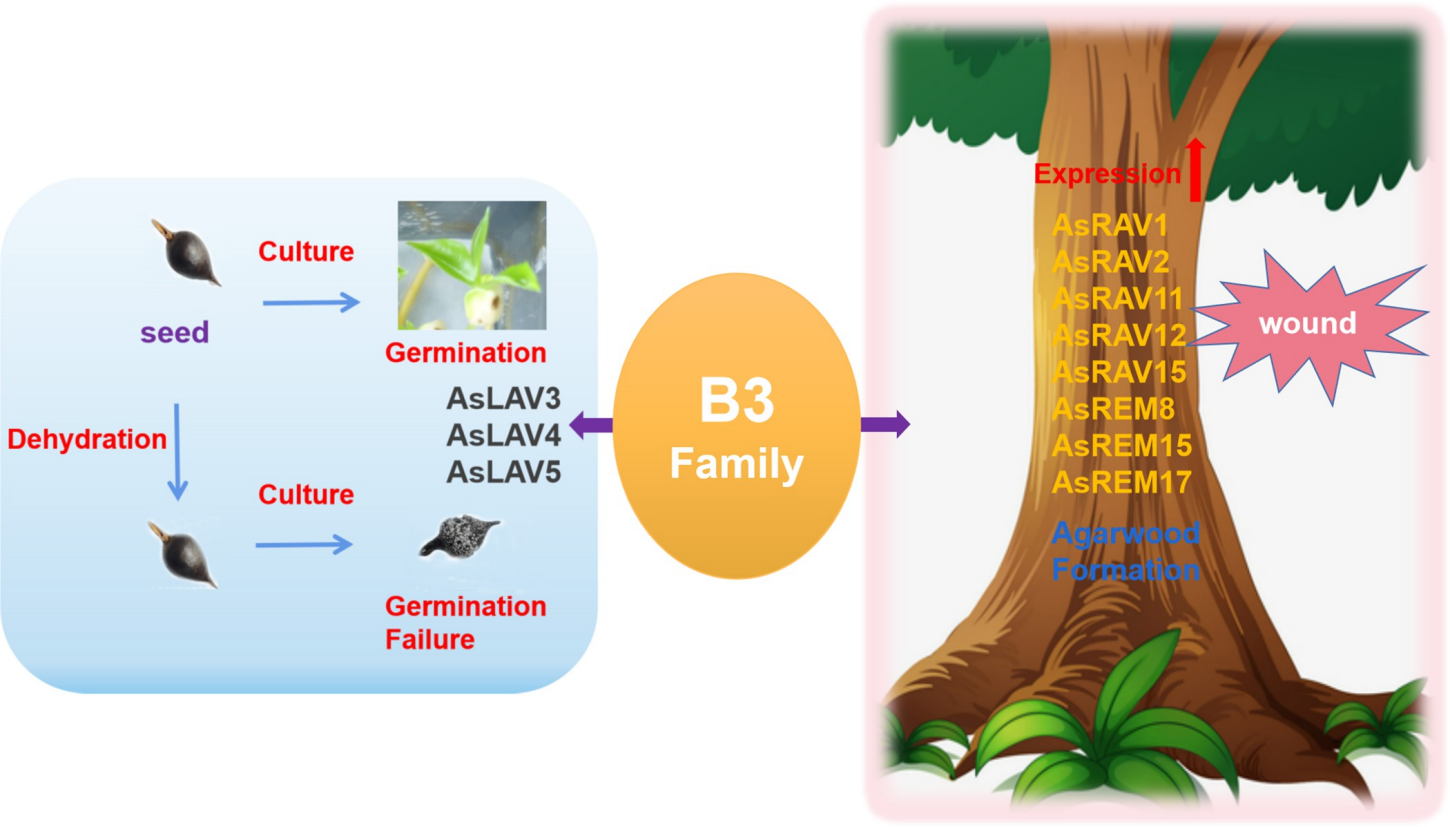
A summary of the B3 superfamily and their possible involvement in seed recalcitrance and agarwood formation.

In our RNA-seq results, the *AFL* subfamily genes were almost not expressed in any of the tested tissue; this is highly consistent with previous work that showed that the expression of *AFL* members in *Arabidopsis* and rice was seed specific [13]. To find more factors leading to the low desiccation tolerance, we used qRT-PCR to compare the expression levels of candidate *AFL* genes in leaves, flowers, seeds, and fruits. Unexpectedly, the LEC2 ortholog was not expressed in seeds. A study in *Arabidopsis* showed that ectopic expression of LEC2, FUS3, or ABI3 in the single- or double-mutant backgrounds of the other two regulators could not initiate the desiccation tolerance of seed, suggesting that all three regulators are required to activate desiccation tolerance [24]. These findings indicate that the absence of LEC2 expression may be related to the low desiccation tolerance (Fig 7). The clear relationship needs further investigation, including the detailed examination of the expression of *AFL* genes in different tissues and different seed developmental stages at both transcriptional and posttranscriptional levels as well as the function confirmation with genetic approaches. Conversely, a series of studies in *Arabidopsis*, rice, and grape have showed that the *VAL* genes were relatively ubiquitously expressed throughout plant development [23, 25–27]. Our results also support this finding. Since VALs have been proved to be inhibitors of *AFL* genes [25, 28–30], their high-level expression in a series of our tested tissues from adult plants might contribute to the low level of expression levels of *AFL* genes in these organs.

The *ARF* genes belong to a well-studied family. In our research, all the *ARF* family genes were expressed at comparative high levels in tested tissues. This finding is slightly different from previous studies which showed that several *ARF* genes were flower specific [5, 22] while several members of the *ARF* gene family play roles in floral organ maturation [31, 32] or abscission [33, 34] in *Arabidopsis*, rice, or tobacco. This may indicate species-specific functions of the *ARF* genes.

Previous work found that many *REMs* were mainly expressed in the shoot apex [35]. Our observation of the high expression levels of *REMs* in buds is consistent with this, suggesting the role of *REMs* in the shoot meristem. Many *RAVs* were expressed at a low level or not expressed in the tested tissues from adult plants, and the expression of the other *RAVs* was tissue specific, and this is consistent with a study in *Arabidopsis* [13]. Furthermore, the expression patterns of these *RAVs* were diverse, indicating that unlike *ARF* genes with redundant functions [36, 37], these genes may have different functions in the development of *A*. *sinensis*.

We found that a series of *B3* genes, including the *RAV* and *REM* members (Fig 7), could be induced by wound treatment, and their expression patterns were consistent with those of *TPS* genes (unpublished results). The RAV family members, AaERF1 or AaERF2, can elevate transcript levels of amarpha-4,11-diene synthase, a sesquiterpene synthase, thereby increasing accumulation of artemisinin and artemisinic acid in *A*. *annua*. RAV1 was shown by coexpression analysis to regulate the *HMGR* gene as well as epoxysqualene biosynthesis and sterol biosynthesis in *Arabidopsis* [16]. Here, although several RAV members were found to be wound-responsive in this research, none of them contains the AP2 domain, which is present in RAV1. Moreover, very little is known about the function of the plant-specific B3 TFs in the wound or biotic stress responses. Therefore, the relationship of these wound-induced B3 TFs in the production of secondary metabolites in agarwood and in the wound or biotic stress responses requires further investigation.

We also identified 14 *A*. *sinensis*-specific RAV members which clustered in a single clade in the phylogenetic tree. Similar species-specific clades have also been shown in other studies, although they have not been sufficiently discussed [23, 38]. Our study showed that these AsRAVs has similar motifs with some differences compared to other AsRAVs. Importantly, several of them were wound responsive. These demonstrated that these species-specific genes might participate in the species-specific function of *A*. *sinensis*. These results provides newly clues for our future work.

## 4. Materials and methods

### 4.1. Plant materials and treatment

Tissue samples (root, stem, branch, new leaf, old leaf, bud, flower, agarwood, seed, and fruit) were collected from the trees of *A*. *sinensis* growing in the YanFeng Basement of Hainan Branch, Institute of Medicinal Plant Development (IMPLAD). These materials were treated and collected with permission. Plants were identified by Prof. JianHe Wei and Dr. Lin Zeng, and the vouched specimen (469023-CM-044) was deposited in the herbarium and the National Southern Medicine Gene Bank of China in Hainan Branch, IMPLAD.

For the wound treatments, healthy *A*. *sinensis* trees cultivated in the YanFeng Basement of Hainan Branch, IMPLAD were selected. A patent-protected chemical-injection technique (Agar-Wit) was conducted on stems 60–70 mm in diameter. For samples at 2, 6, 12, and 24 h, and 3, 5, and 15 d, treated plant materials were collected in the stem sections 20–50 cm above the injected sites (with no branch in this section). To facilitate the identification of the different layers, Prussian blue was used to mark the D layer (Dead layer). The agarwood and transition layer (AT layer) was the brown layer outside of the D layer (~1cm thick). The healthy layer (H layer) is the white material outside the AT layer.

The anatomy of *A*. *sinensis* agarwood undergoes significant changes by 21 m after AgarWIT treatment: whereas the newly treated trees have combined A and T layers, this is clearly two layers by 21 months (the A and the T layer). For the 21-m samples, the T layer is darker than the H layer yet lighter than the A layer. The block layer (B layer) was the newly formed white layer between the A and T layers. Three biological replicates were collected for each sample.

### 4.2. Desiccation of seeds and germination test

To test the effect of dehydration on seed germination, 25 seeds were placed in containers and cultured at 25°C and 20% relative humidity, giving four replicates for each of the three target moisture contents (TMCs) of 40%, 30%, and 20%. The moisture content was calculated by the following formula: (Weight of fresh seeds−Weight of dry seeds) / Weight of fresh seeds × 100%.

For the germination test with peeled and unpeeled seeds, 25 or 50 seeds were placed in plastic boxes immediately after the seeds had arrived from Hainan province. For the germination test with desiccated seeds, each test was replicated with 25 seeds. The germination test was initiated immediately when the seeds had reached the target TMC. Incubation boxes were then placed in an incubator with a light regime of 8/16 h at 25°C. The germination test was terminated after 12 days.

### 4.3. Identification and classification of B3 genes in the *A*. *sinensis* genome

Identification of *B3* genes was based on our whole-genome protein sequence of *A*. *sinensis*, which has been uploaded to the NCBI (PRJNA663400). A HMM profile of the B3 domain (PF02362) was obtained from the Pfam database (http://pfam.sanger.ac.uk). HMMER software with the conserved B3 domain and default parameters was adopted to search for B3 protein sequences. Then SMART database (http://smart.embl-heidelberg.de/) and NCBI CD-search tools (http://www.ncbi.nlm.nih.gov/Structure/cdd/wrpsb.cgi) were used to verify the reliability of those candidate B3 sequences. Redundant sequences or sequences lacking the B3 domain were removed.

ExPASy ProtParam program (https://web.expasy.org/protparam/) was used to predict the pI and molecular weight. The Protein Subcellular Localization Prediction Tool PSORT (https://www.genscript.com/psort.html) was used for the prediction of subcellular localizations.

B3 family members were named according to their subfamily, after being classified by the domain analysis and phylogenetic analysis. The number in the name was assigned according to the sequence of their gene ID from the genome annotation (S1 Table).

### 4.4. Phylogenetic analysis

In total, 68 sequences of AtB3 proteins were downloaded from the Arabidopsis database (TAIR, http://www.arabidopsis.org/). Sequences from *A*. *sinensis* and Arabidopsis were aligned using Clustal X. Phylogenetic analysis was then conducted using IQ-TREE (http://iqtree.cibiv.univie.ac.at/) with the maximum likelihood method (Jones Taylor Thornton model+R4+F), and bootstrap values were calculated using 1000 repetitions (ultrafast mode). The phylogenetic tree was visualized using the Interactive Tree of Life (iTOL) [39].

### 4.5. Gene structure, conserved motif, chromosomal location, and gene duplication

The exon and intron organizations of *AsB3* genes were determined by comparing the coding sequence with their corresponding genomic sequences using the GSDS tool (http://gsds.cbi.pku.edu.cn/). Structural motif annotation was performed using the MEME program (http://meme-suite.org/). PHD-L domains were identified by protein sequence alignment using DNAMAN. The physical distribution of the *AsB3* genes on chromosomes was drawn using MapDraw. The identification of the conserved motifs was performed using the MEME program (http://meme-suite.org/). Tandem duplications were detected using the method described by Guo et al. [40], with the following criteria: (1) the length of the sequence alignment covered ≥70% of the longest gene; (2) similarity of the aligned regions was ≥70% and the distance between genes was <100 kb. Segmental duplication events were calculated using MCScanX with default parameters [41].

### 4.6. Expression pattern of B3 genes

Materials used in the RNA-seq have been described in section 4.1. The RNA-seq data for tissue-specific analysis and induced expression analysis were also derived from data (BioProject number PRJNA663400) submitted to NCBI. Transcript sequences were aligned to the genome with Tophat (2.0.11) and Cufflinks (2.1.1). High-throughput RNA-seq data of *A*. *sinensis* for *B3* genes in different tissues and in branches after wound treatment were then used to analyze the expression of *B3* genes by the reads per kilobase per million reads mapped (RPKM) method. To visualize the expression pattern of *B3* genes after wound treatments, the expression data of all wound-treated samples were standardized and then processed to generate the Heat Map using Heatmapper [42] with the y = log_2_ + [(Trpkm + 1)/(Crpkm + 1)] as the ordinate, where Trpkm indicates the rpm value of the treated group, and Crpkm indicates the rpm value of the control group H0H (untreated sample, H layer at 0 h after the treatment). The color range was a uniform gradient change, from orange to blue (high to low). For the tissue samples, the heatmap was made with log_2_ (RPKM+1).

For the qRT-PCR, total RNA was extracted from 100 mg different tissues using a Total RNA Rapid Extraction kit (Aidlab, RN38-EASYspin Plus). The EasyScript® One-Step gDNA Removal and cDNA Synthesis SuperMix kit (TransGen, AE311) was used to synthesize single-stranded cDNA from 1 μg total RNA according to the manufacture’s protocol. qRT-PCR was performed with the TransStart® Top Green qPCR SuperMix kit (TransGen, AQ131). *AsTUA* as internal control [43] and the 2^-ΔΔCT^ method were used to calculate the relative expression levels of genes with three biological replicates and three technical replicates.

### 4.7. Ethical approval

All procedures were conducted in accordance with the relevant institutional, national, and international guidelines and legislation.

## 5. Conclusions

The functions of the B3 TF superfamily, especially in areas other than plant development, remain largely unknown. In this work, the members of the B3 superfamily were identified in *A*. *sinensis* and classified into different subfamilies by phylogenetic and domain structure analysis. The motif analysis showed that each subfamily did not only have conserved domains, but that the B3 domain contained conserved motifs in the B3 domain. The observations of the protein structure in the VAL subfamily and the expression pattern of *AsLAV4-*the *LEC2* ortholog provides guidance for future studies in the low desiccation tolerance of the recalcitrant seeds of *A*. *sinensis*. Fourteen *A*. *sinensis*-specific RAVs were identified with special protein structure and several of them could be induced by wound. Several other *RAVs* and *REMs* induced after wound treatment were also identified, indicating a possible role of these *B3* genes in wound response and agarwood formation.

## Supporting information

S1 Table Characteristics of B3 genes in *A*. *sinensis*. (DOCX)

S2 Table Information of Arabidopsis B3. (XLSX)

S1 Fig Phylogenetic tree of AsLAV proteins. The phylogenetic tree was constructed with the MEGA 7.0 program by the maximum liklyhood method.
(TIFF)

S2 Fig Sequence logo for the ten conserved motifs, identified using MEME tools, from the B3 proteins in *A*. *sinensis*. The height of residues within the stack indicates the probability of each residue.
(TIF)

S3 Fig PHD-L domain in AsLAV proteins. (TIF)

S4 Fig Germination results of the fresh seeds and dried seeds. (A) fresh peeled seed; (B) seedlings growth of the fresh seeds after being cultured for 12 days; (C) unpeeled dried seeds;(D) unpeeled dried seeds after being cultured for 15 days.
(TIF)

S1 File AsB3 protein sequences. (TXT)

S2 File AsB3 CDS sequences. (TXT)
