# Supplementary material for: Exploration of the B3 transcription factor superfamily in Aquilaria sinensis reveal their involvement in seed recalcitrance and agarwood formation
Source: PLoS One. 2023 Nov 16;18(11):e0294358. doi: 10.1371/journal.pone.0294358 (PMC10653465; doi:10.1371/journal.pone.0294358)
Supplement: S1 Table — (DOCX) [file pone.0294358.s001.docx]

**S1 Table. Characteristics of B3 genes in *A. sinensis***

| **Gene Name** | **Gene ID** | **CDS size** | **No. of exons** | **Protein size** | **subfamily** | **PI** | **MW** | **location** | **B3** | **Auxin_resp** | **AUX_IAA** | **zf-CW** |
| --- | --- | --- | --- | --- | --- | --- | --- | --- | --- | --- | --- | --- |
| AsARF1 | >evm.model.scaffold10133.7 | 1995 | 12 | 664 | ARF | 5.94 | 72826.39 | nuclear | 149-251 | 275-357 |  |  |
| AsARF2 | >evm.model.scaffold107503.82 | 2145 | 14 | 714 | ARF | 6.62 | 79111.52 | nuclear | 126-228 | 252-331 | 567-639, 635-689 | |
| AsARF3 | >evm.model.scaffold109403.25 | 2028 | 14 | 675 | ARF | 5.94 | 74833.25 | nuclear | 127-229 | 253-335 | 520-642 |  |
| AsARF4 | >evm.model.scaffold113599.17 | 1743 | 2 | 580 | ARF | 5.91 | 63236.34 | mitochondrial | 118-220 | 266-349 |  |  |
| AsARF5 | >evm.model.scaffold132565.17 | 2217 | 10 | 738 | ARF | 6.54 | 81013.14 | nuclear | 155-257 | 281-363 |  |  |
| AsARF6 | >evm.model.scaffold134631.58 | 2109 | 3 | 702 | ARF | 6.24 | 76683.84 | cytoplasmic | 121-223 | 290-373 |  |  |
| AsARF7 | >evm.model.scaffold33015.4 | 3339 | 13 | 1112 | ARF | 5.96 | 123713.25 | nuclear | 121-223 | 247-329 | 952-1074 |  |
| AsARF8 | >evm.model.scaffold43899.12 | 2025 | 3 | 674 | ARF | 6.27 | 74289.59 | nuclear | 121-223 | 282-365 |  |  |
| AsARF9 | >evm.model.scaffold54207.28.6 | 2238 | 15 | 745 | ARF | 8.16 | 83505.57 | nuclear | 156-258 | 281-362 | 672-723 |  |
| AsARF10 | >evm.model.scaffold56815.28 | 2829 | 14 | 942 | ARF | 5.84 | 103031.72 | nuclear | 150-252 | 276-359 | 787-918 |  |
| AsARF11 | >evm.model.scaffold7677.39 | 3417 | 14 | 1138 | ARF | 6.39 | 126017.29 | nuclear | 140-241 | 265-347 | 1009-1108 |  |
| AsARF12 | >evm.model.scaffold87555.9 | 2061 | 14 | 686 | ARF | 6.03 | 76977.13 | nuclear | 126-228 | 252-331 | 535-663 |  |
| AsLAV1 | >evm.model.scaffold104821.12 | 2733 | 13 | 910 | LAV | 5.67 | 99788.64 | nuclear | 317-417 |  |  | 586-629 |
| AsLAV2 | >evm.model.scaffold112873.29 | 2523 | 12 | 968 | LAV | 7.44 | 106205.18 | nuclear | 335-435 |  |  | 566-609 |
| AsLAV3 | >evm.model.scaffold125525.10 | 2412 | 12 | 250 | LAV | 6.11 | 28690.56 | nuclear | 128-233 |  |  |  |
| AsLAV4 | >evm.model.scaffold131807.3 | 1625 | 7 | 407 | LAV | 8.18 | 45655.89 | nuclear | 173-274 |  |  |  |
| AsLAV5 | >evm.model.scaffold31049.24 | 1570 | 5 | 582 | LAV | 5.77 | 64831.96 | nuclear | 29-129 |  |  | 294-337 |
| AsLAV6 | >evm.model.scaffold32371.13 | 275 | 5 | 293 | LAV | 5.76 | 32987.95 | nuclear | 105-207 |  |  |  |
| AsLAV7 | >evm.model.scaffold86967.64 | 197 | 1 | 703 | LAV | 6.27 | 78375.89 | nuclear | 554-656 |  |  |  |
| AsRAV1 | >evm.model.scaffold103857.10 | 789 | 3 | 262 | RAV | 7.28 | 29853.36 | nuclear | 52-155 |  |  |  |
| AsRAV2 | >evm.model.scaffold125647.54 | 1529 | 7 | 326 | RAV | 7.72 | 36303.53 | nuclear | 77-173 |  |  |  |
| AsRAV3 | >evm.model.scaffold126581.33 | 1124 | 4 | 139 | RAV | 9.54 | 15910.48 | cytoplasmic | 23-120 |  |  |  |
| AsRAV4 | >evm.model.scaffold130013.39 | 1622 | 7 | 204 | RAV | 9.46 | 23537.23 | nuclear | 4-102 |  |  |  |
| AsRAV5 | >evm.model.scaffold130281.4 | 1625 | 7 | 92 | RAV | 9.3 | 10779.27 | cytoplasmic | 2-91 |  |  |  |
| AsRAV6 | >evm.model.scaffold136531.87 | 1377 | 6 | 140 | RAV | 9.89 | 15656.36 | cytoplasmic | 5-118 |  |  |  |
| AsRAV7 | >evm.model.scaffold136531.88 | 1376 | 7 | 117 | RAV | 6.41 | 13223.16 | cytoplasmic/ nuclear | 4-102 |  |  |  |
| AsRAV8 | >evm.model.scaffold136875.35 | 1270 | 7 | 118 | RAV | 8.85 | 13719.75 | nuclear | 5-106 |  |  |  |
| AsRAV9 | >evm.model.scaffold141953.22 | 1805 | 7 | 168 | RAV | 10.48 | 19169.02 | nuclear | 34-130 |  |  |  |
| AsRAV10 | >evm.model.scaffold141953.23 | 971 | 4 | 150 | RAV | 10.57 | 16957.88 | cytoplasmic | 17-112 |  |  |  |
| AsRAV11 | >evm.model.scaffold142139.4 | 967 | 5 | 381 | RAV | 6.43 | 42788.59 | nuclear | 10-205 |  |  |  |
| AsRAV12 | >evm.model.scaffold16559.33 | 867 | 7 | 115 | RAV | 9.99 | 12823.36 | cytoplasmic | 5-102 |  |  |  |
| AsRAV13 | >evm.model.scaffold16559.35 | 1559 | 7 | 122 | RAV | 7.11 | 13984.97 | cytoplasmic | 4-106 |  |  |  |
| AsRAV14 | >evm.model.scaffold40873.36 | 878 | 4 | 116 | RAV | 9.38 | 13361.24 | mitochondrial | 4-103 |  |  |  |
| AsRAV15 | >evm.model.scaffold47097.14 | 568 | 2 | 324 | RAV | 6.83 | 35768.12 | nuclear | 48-153 |  |  |  |
| AsRAV16 | >evm.model.scaffold74403.58 | 174 | 1 | 119 | RAV | 6.9 | 13408.35 | cytoplasmic | 4-103 |  |  |  |
| AsRAV17 | >evm.model.scaffold99573.1 | 822 | 3 | 108 | RAV | 6.82 | 12755.44 | cytoplasmic | 5-103 |  |  |  |
| AsRAV18 | >evm.model.scaffold99573.6 | 795 | 3 | 117 | RAV | 9 | 13475.48 | nuclear | 4-103 |  |  |  |
| AsREM1 | >evm.model.scaffold109265.31 | 2481 | 15 | 226 | REM | 10.22 | 25827.91 | nuclear | 13-106 |  |  |  |
| AsREM2 | >evm.model.scaffold109265.32 | 2550 | 15 | 258 | REM | 8.51 | 29987.54 | cytoplasmic | 8-99, 153-247 | |  |  |
| AsREM3 | >evm.model.scaffold109265.33 | 2344 | 14 | 267 | REM | 9.82 | 30198.51 | cytoplasmic | 8-99, 170-263 | |  |  |
| AsREM4 | >evm.model.scaffold117607.134 | 2520 | 12 | 374 | REM | 8.52 | 42341.35 | cytoplasmic | 25-115,253-360 | |  |  |
| AsREM5 | >evm.model.scaffold12813.6 | 1706 | 7 | 304 | REM | 8.84 | 34839.48 | cytoplasmic | 14-108, 201-302 | |  |  |
| AsREM6 | >evm.model.scaffold129195.22 | 1422 | 6 | 153 | REM | 9.84 | 17740.78 | cytoplasmic | 21-113 |  |  |  |
| AsREM7 | >evm.model.scaffold136627.15 | 232 | 2 | 189 | REM | 9.01 | 22112.71 | cytoplasmic | 58-153 |  |  |  |
| AsREM8 | >evm.model.scaffold136627.17 | 253 | 2 | 406 | REM | 9.08 | 46598.92 | nuclear | 27-117,290-387 | |  |  |
| AsREM9 | >evm.model.scaffold136627.18 | 661 | 5 | 364 | REM | 9.67 | 42371.72 | cytoplasmic | 27-118,247-350 | |  |  |
| AsREM10 | >evm.model.scaffold140017.19 | 2400 | 12 | 408 | REM | 9.35 | 47712.68 | nuclear | 27-117,288-389 | |  |  |
| AsREM11 | >evm.model.scaffold16559.29 | 1651 | 7 | 287 | REM | 5.24 | 32571.55 | cytoplasmic | 44-137,195-283 | |  |  |
| AsREM12 | >evm.model.scaffold16559.30 | 2835 | 12 | 324 | REM | 9.12 | 37118.62 | mitochondrial | 6-98,227-322 | |  |  |
| AsREM13 | >evm.model.scaffold16559.31 | 1736 | 7 | 338 | REM | 9.3 | 38525.23 | nuclear | 25-118,244-337 | |  |  |
| AsREM14 | >evm.model.scaffold16559.32 | 1823 | 7 | 340 | REM | 9.6 | 38637.04 | nuclear | 25-118,246-339 | |  |  |
| AsREM15 | >evm.model.scaffold24595.7 | 1805 | 7 | 307 | REM | 9.42 | 35100.63 | nuclear | 18-112,211-305 | |  |  |
| AsREM16 | >evm.model.scaffold34921.101 | 1570 | 5 | 410 | REM | 9.02 | 46221.2 | mitochondrial | 52-144,304-399 | |  |  |
| AsREM17 | >evm.model.scaffold34921.104 | 438 | 3 | 324 | REM | 8.99 | 37370.72 | mitochondrial | 6-98,226-321 | |  |  |
| AsREM18 | >evm.model.scaffold34921.116 | 835 | 5 | 279 | REM | 10.13 | 31769.83 | mitochondrial | 177-268 |  |  |  |
| AsREM19 | >evm.model.scaffold45451.11 | 223 | 2 | 313 | REM | 9.78 | 35332.71 | nuclear | 142-233 |  |  |  |
| AsREM20 | >evm.model.scaffold46899.17 | 309 | 3 | 386 | REM | 8.42 | 43720.84 | cytoplasmic | 24-115,257-350 | |  |  |
| AsREM21 | >evm.model.scaffold46899.18 | 232 | 2 | 576 | REM | 8.78 | 64857.04 | extracellular | 9-100,256-352,469-565 | |  |  |
| AsREM22 | >evm.model.scaffold47097.158 | 598 | 2 | 470 | REM | 9.54 | 52807.74 | nuclear | 18-112,239-340,373-468 | |  |  |
| AsREM23 | >evm.model.scaffold47097.159_ [TF]B3:B3_superfamily->B3--NA | 411 | 3 | 532 | REM | 8.89 | 60202.54 | cytoplasmic | 93-188,293-387,431-522 | |  |  |
| AsREM24 | >evm.model.scaffold613520.1.1 | 579 | 3 | 364 | REM | 9.61 | 41242.88 | nuclear | 1-60,170-271 | |  |  |
| AsREM25 | >evm.model.scaffold81999.7 | 296 | 1 | 318 | REM | 9.34 | 36402 | mitochondrial | 62-153,215-311 | |  |  |
| AsREM26 | >evm.model.scaffold82777.3 | 232 | 2 | 808 | REM | 6.58 | 91993.67 | cytoplasmic | 139-230,445-536 | |  |  |
| AsREM27 | >evm.model.scaffold85295.14.1 | 332 | 1 | 338 | REM | 8.44 | 38696.09 | mitochondrial | 39-133,239-338 | |  |  |
| AsREM28 | >evm.model.scaffold91791.27 | 265 | 2 | 289 | REM | 6.55 | 33304.37 | cytoplasmic | 14-105,194-289 | |  |  |
| AsREM29 | >evm.model.scaffold91791.29 | 293 | 1 | 210 | REM | 9.14 | 24068.53 | cytoplasmic | 116-210 |  |  |  |
| AsREM30 | >evm.model.scaffold91791.30 | 272 | 1 | 261 | REM | 9.79 | 29810.67 | mitochondrial | 11-102,164-259 | |  |  |
| AsREM31 | >evm.model.scaffold96371.5 | 170 | 1 | 255 | REM | 6.11 | 29055.88 | cytoplasmic | 12-108,157-246 | |  |  |
| AsRAV19 | >evm.model.scaffold128681.25 | 1053 | 1 | 350 | RAV | 6.22 | 39818.74 | cytoplasmic | 166-273 | 43-101 |  |  |
| AsRAV20 | >evm.model.scaffold139881.12 | 1044 | 1 | 347 | RAV | 9.02 | 39649.72 | cytoplasmic | 156-250 | 37-95 |  |  |
| AsRAV21 | >evm.model.scaffold612436.1 | 1056 | 1 | 351 | RAV | 8.93 | 39410.19 | nuclear | 190-294 | 64-119 |  |  |
