# Supplementary figures and images for: Exploration of the B3 transcription factor superfamily in Aquilaria sinensis reveal their involvement in seed recalcitrance and agarwood formation
Source: PLoS One. 2023 Nov 16;18(11):e0294358. doi: 10.1371/journal.pone.0294358 (PMC10653465; doi:10.1371/journal.pone.0294358)

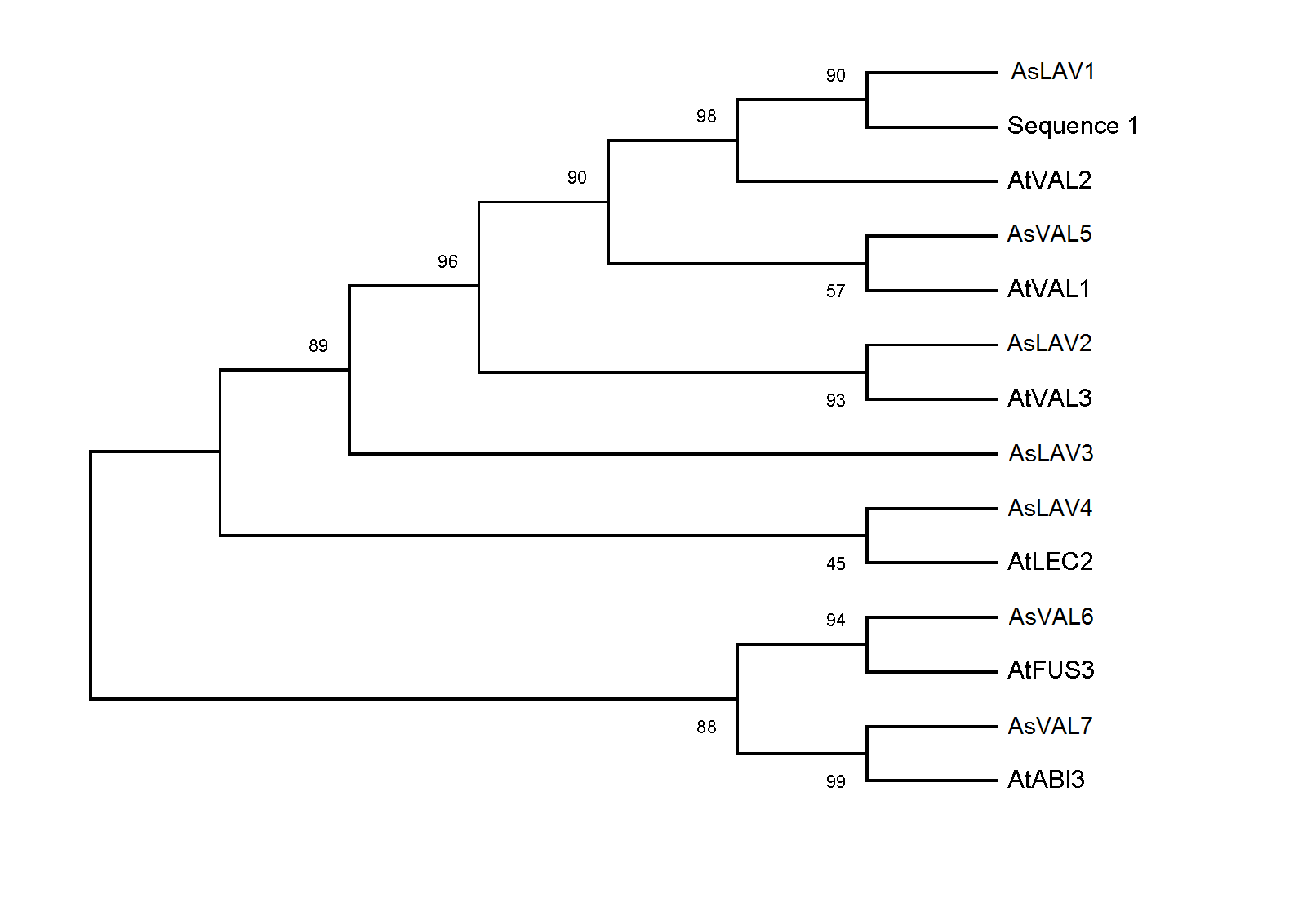

Supplement: S1 Fig — The phylogenetic tree was constructed with the MEGA 7.0 program by the maximum liklyhood method. (TIFF) [file pone.0294358.s003.tiff]

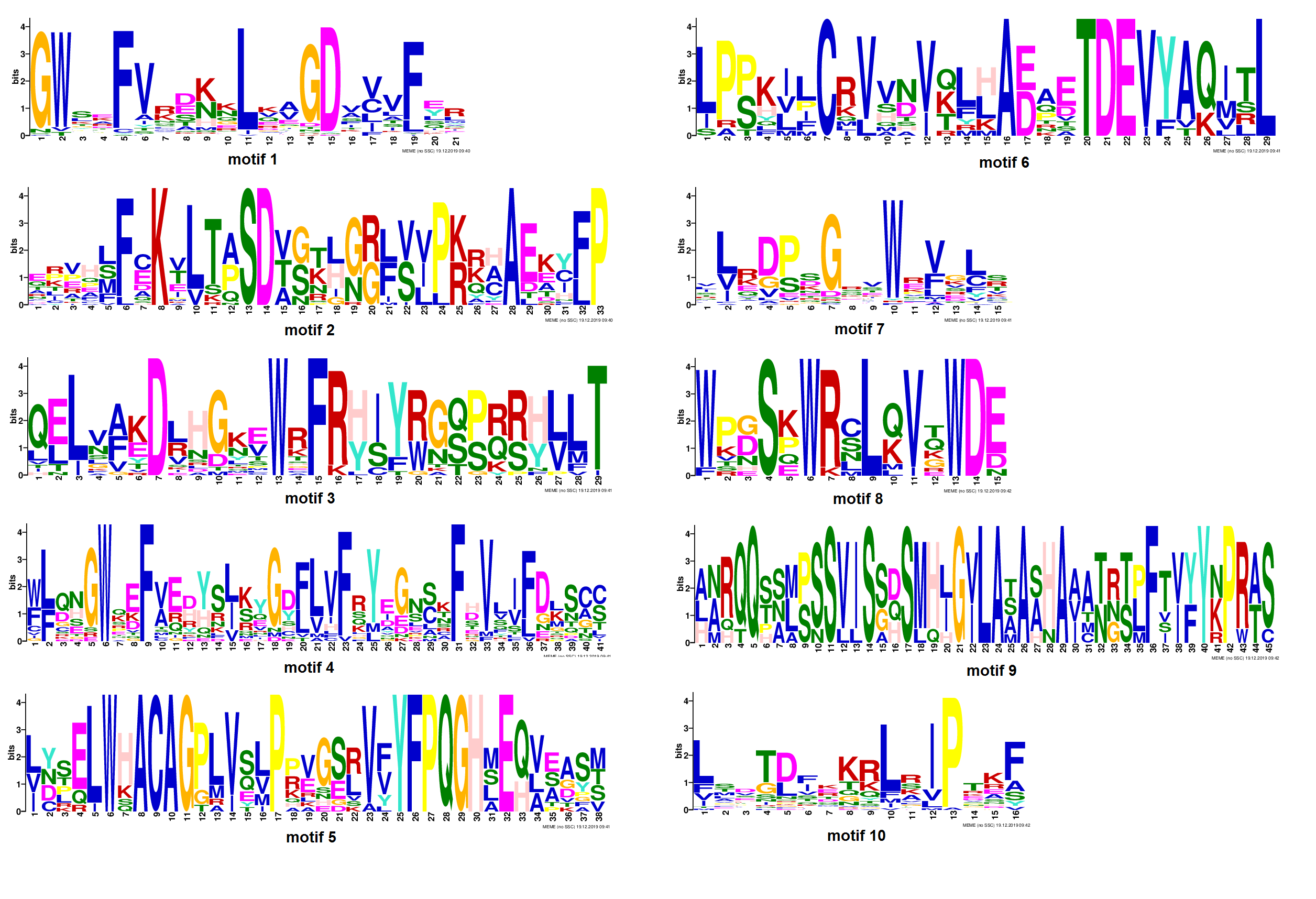

Supplement: S2 Fig — The height of residues within the stack indicates the probability of each residue. (TIF) [file pone.0294358.s004.tif]

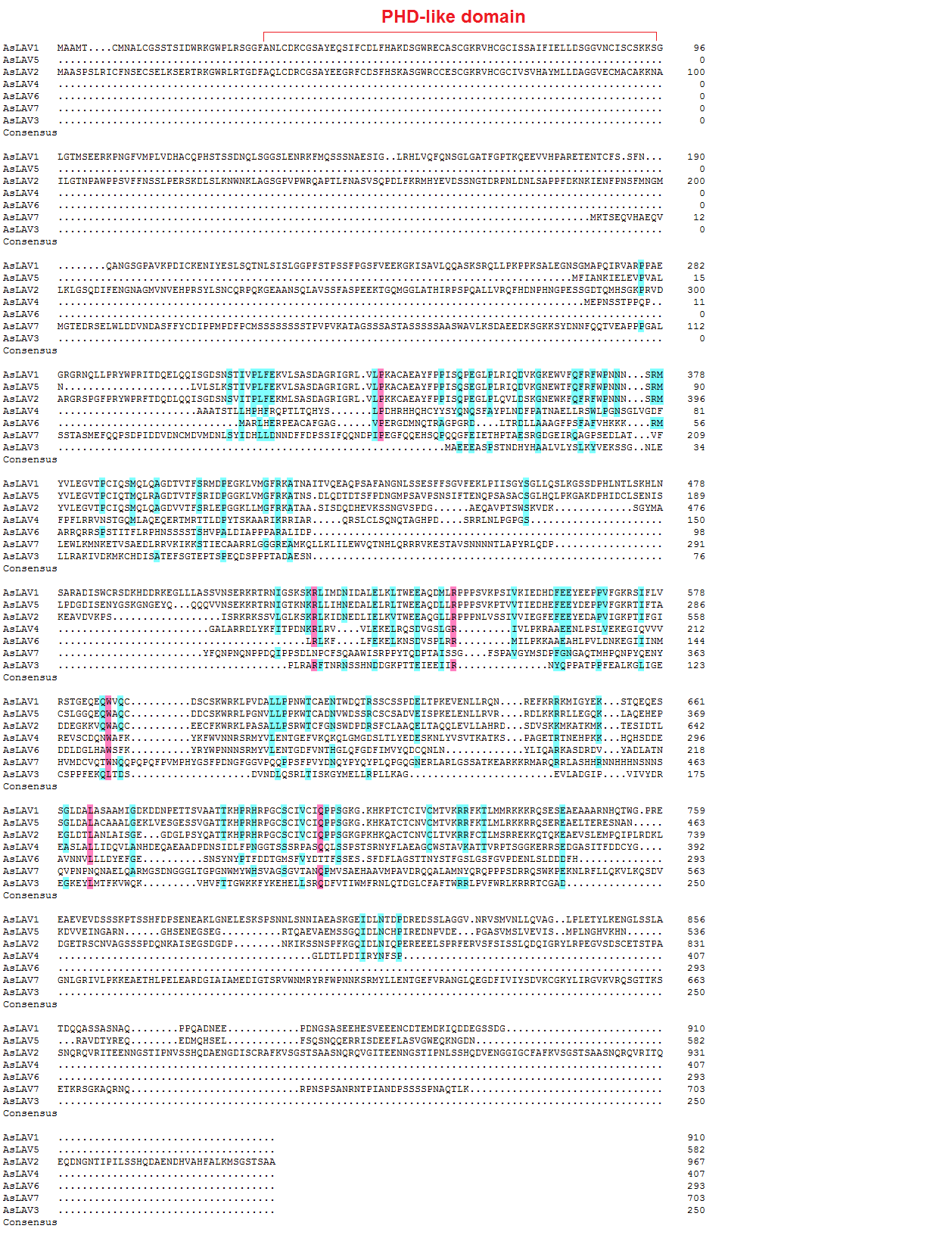

Supplement: S3 Fig — (TIF) [file pone.0294358.s005.tif]

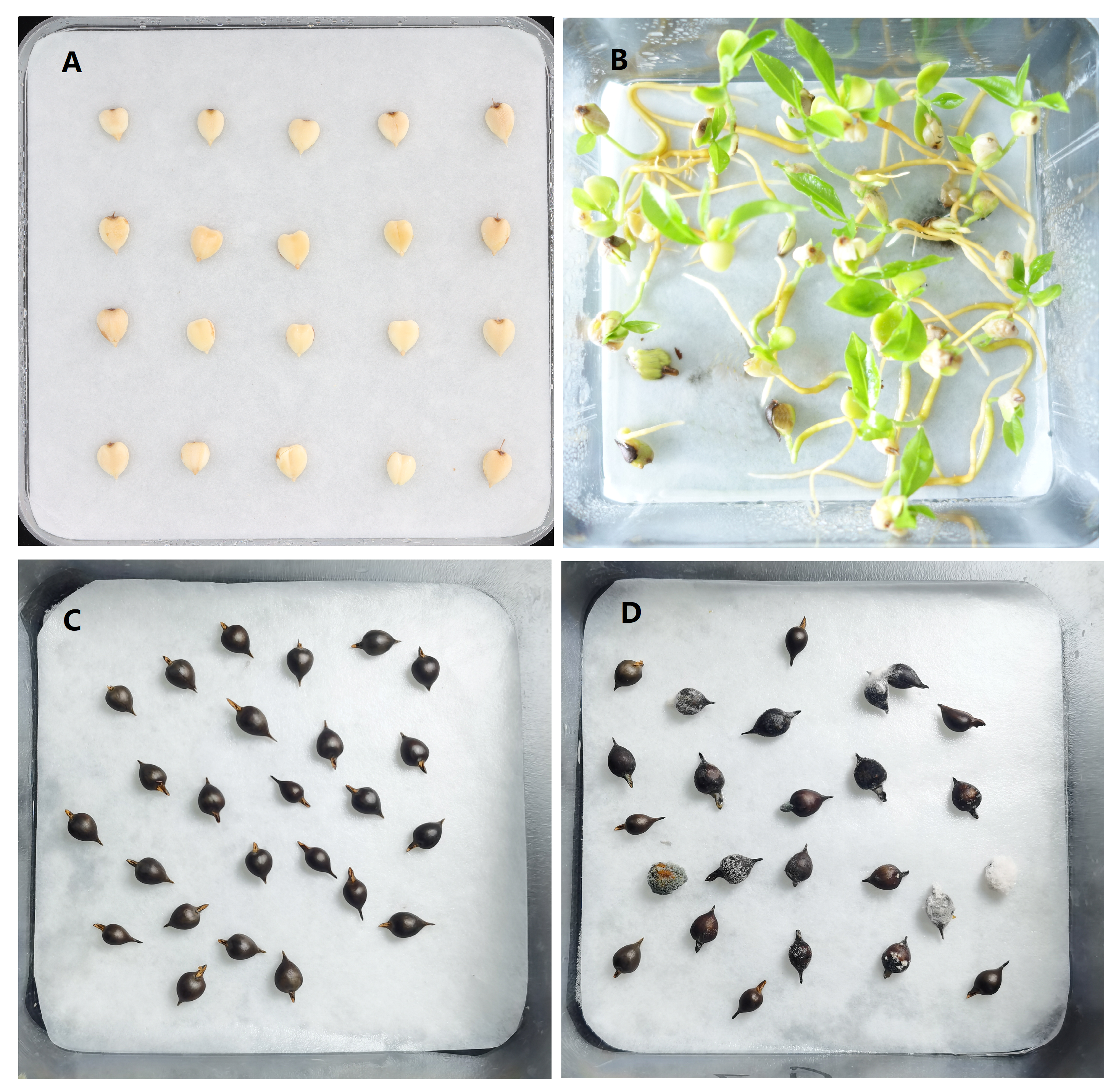

Supplement: S4 Fig — (A) fresh peeled seed; (B) seedlings growth of the fresh seeds after being cultured for 12 days; (C) unpeeled dried seeds;(D) unpeeled dried seeds after being cultured for 15 days. (TIF) [file pone.0294358.s006.tif]
